# Characterization of Acute Myeloid Leukemia With RUNX1/RUNX1T1 Gene Rearrangement: Clinical, Hematological, and Morphological Features

**DOI:** 10.7759/cureus.74760

**Published:** 2024-11-29

**Authors:** Sadaf Maqbool, Iqra Maqbool, Marya Yousaf, Birya Farooqi, Mirza Zeeshan Sikandar, Ridha Zainab, Khush Bakht, Mishal Shahid

**Affiliations:** 1 Internal Medicine, Egon German Clinic Accra, Accra, GHA; 2 Internal Medicine, Akhter Saeed Medical and Dental College, Lahore, PAK; 3 Community Medicine, Central Park Medical College, Lahore, PAK; 4 General Surgery, Sargodha Medical College, Sargodha, PAK; 5 Nephrology, Central Park Medical College, Lahore, PAK; 6 Oncology, Aziz Bhatti Shaheed Teaching Hospital, Gujrat, PAK; 7 Psychiatry, Lincolnshire Partnership NHS Foundation Trust, London, GBR; 8 Oncology, Pak Red Cresent Medical And Dental College, Lahore, PAK

**Keywords:** acute myeloid leukemia (aml), french-american-british (fab), gene rearragement, polymerase chain reaction (pcr), runt-related transcription factor 1 (runx1)

## Abstract

Objectives: This study aimed to determine the frequency of RUNX1/RUNX1T1 gene rearrangement in acute myeloid leukemia (AML) patients by polymerase chain reaction (PCR) and analyze their clinical, hematological, and morphological features of positive patients.

Patients and methods: A cross-sectional study was conducted in which newly diagnosed patients with AML were included in the study. A total of 101 AML cases were calculated from the World Health Organization (WHO) formula. Sysmex Hematology Analyzer XP-100 (Sysmex Corporation, Kobe, Japan) performed a complete blood picture of these positive patients. Molecular analysis was carried out by reverse transcriptase-polymerase chain reaction (RT-PCR).

Results: A total of 101 AML cases were enrolled. Twelve (11.9%) were found positive for this specific recurrent RUNX1/RUNX1T1 gene rearrangement. Nine (75%) were males, while three (25%) were females. The mean age of the participants was 42 years. The most common clinical feature was pallor. The average count of hemoglobin, platelets, and total leukocyte count was 8 g/dl, 41.5×10^9^/L, and 71.4×10^9^/L, respectively. Bone marrow aspiration showed erythropoiesis, thrombopoiesis, and myelopoiesis depressed in all positive cases of acute myeloblastic leukemia with maturation (AML-M2). The mean blast percentage of AML-M2 was 58%. Auer rods were also found in these positive patients.

Conclusion: Identifying this fusion protein in AML patients with the AML-M2 FAB subtype is valuable because it has prognostic and therapeutic significance.

## Introduction

The diverse collection of hematological cancers known as acute myeloid leukemia (AML) is defined by the clonal proliferation of immature myeloid progenitors (blasts) in the bone marrow [1]. About 4.2 instances per 100,000 people are recorded each year in both men and women. In the US, there are more than 20,000 cases of AML annually. In children and young adults with AML, this RUNX1-RUNX1T1 gene rearrangement is 15%-25% common [2]. The age at which AML presentation occurs reveals that it peaks twice, first in infancy and again around the age of 60. The frequency of the condition rises with age, particularly in males over 65 [3]. Males are reported to have the illness at a higher rate than females [4].

A transcriptional regulator crucial to myeloid development and hematopoietic differentiation is RUNX1. A transcriptional co-repressor, the RUNX1T1 gene is a member of the human RUNX1T1 homolog family. It needs transcription factors, such as GFI1 and BCL6, to create multi-protein complexes and does not directly bind with DNA [5]. The RUNX1 gene from chromosome 21 and the RUNX1T1 gene from chromosome 8 combine to form a fusion gene as a result of this translocation. There are 752 amino acids in this fusion protein; the first 177 come from RUNX1, while the remaining 575 come from RUNX1T1. Thus, RUNX1-RUNX1T1 comprises five domains structurally: NHR domains 1-4 from RUNX1T1 and the runt-homology domain (RHD) from RUNX1 [6].

Among all AML patients, the most prevalent cytogenetic abnormality is the rearrangement of the RUNX1-RUNX1T1 gene. The patient with bone marrow blasts greater than 20% and peripheral blood blasts greater than 20% is classified as having acute leukemia, according to the World Health Organization (WHO) classification 2022 [7]. Acute myeloid leukemia was once categorized using the fragment antigen binding (FAB) method; however, it is currently categorized using the WHO method. Despite their blast count in the bone marrow or peripheral blood, a small number of AML patients linked to the unique recurring genetic defects t(8;21)(q22;q22), inv(16)(p13q22), t(16;16)(p13;q22), and t(15;17)(q22;q12) should be considered acute leukemia [8].

According to the 2022 WHO classification of AML, particular epigenetic modifications in AML can be found by examining a sample's complete genome or by employing next-generation sequencing (NGS) methods to analyze gene panels, polymerase chain reaction (PCR), or reverse transcriptase-polymerase chain reaction (RT-PCR) tests. Advancements in diagnostic procedures have made it possible to diagnose AML completely and predict its prognosis [9-11]. With an extremely high rate of morbidity and death, it is a potentially lethal illness, and genetic assessment can help in better prognosis and management, but data for the local populace are lacking. Therefore, this study is warranted for the assessment of the frequency of RUNX1/RUNX1T1 gene rearrangement in AML patients by PCR and analyzes the clinical, hematological, and morphological features of positive patients.

## Materials and methods

This multicenteric cross-sectional study was conducted at the Department of Molecular Hematology at Central Park Medical and Teaching Hospital, Lahore, Pakistan, from July 2023 to January 2024 in collaboration with other tertiary care institutions in Pakistan. In this study, newly diagnosed patients with AML were included in the study after getting prior written informed consent, while those patients who were already on treatment were excluded from the study. The sample size was calculated using the WHO calculator [11] by taking the prevalence of AML-ETO at 7% with a confidence interval (CI) of 95% margin of error at 5% and prevalence at 7% of RUNX1 gene in AML patients. The study was conducted after the ethical approval of the Institutional Review Board of Central Park Medical College (approval number: CPMC/IR-no/1486) under the ethical guidelines of the Declaration of Helsinki. 

All the cases were subjected to a detailed clinical history and physical examination, which included the age of presentation, gender, family history, and patient complaints, and physically noted findings on a study proforma. These study symptoms were compared with other symptoms and study variables. Five ccs of venous blood was withdrawn from the medial cubital vein under aseptic conditions after applying a tourniquet at midarm and was stored in ethylenediamine tetraacetic acid (EDTA) vials and was subjected to centrifugation at 4000 rpm for 15 minutes and was sent for hematological and genetic analysis subsequently.

A complete blood picture (CBP) was performed on the Sysmex Hematology Analyzer XP-100 (Sysmex Corporation, Kobe, Japan), and all hematological parameters were noted. Bone marrow aspiration (trephine biopsy) was performed. Peripheral blood and bone marrow smears were stained using Giemsa and Leishman stains according to the WHO criteria of AML [12]. Morphological subtyping of AML was performed according to the FAB criteria of AML-M2 [13]. Molecular analysis was done using the RT-PCR technique. Diagnosis of AML with RUNX1/RUNX1T1 gene rearrangement was also achieved through chromosomal banding analysis, fluorescence in situ hybridization (FISH), and immunophenotyping [14]. For RT-PCR, samples were collected in a vial containing EDTA [15].

Statistical analysis

Anonymized data were entered into a Microsoft Excel spreadsheet, version 2019 (Microsoft Corp., Redmond, WA), and were duly checked and compared for errors and omissions after cross-verification data was imported into IBM SPSS Statistics software, version 26 (IBM Corp., Armonk, NY), and was analyzed. Frequencies and percentages were calculated for qualitative (categorical) variables like gender. Mean ± SD was calculated for quantitative (numerical) variables like age and blood cell counts and was presented in tabular form. The normality of the data was assessed using the Shapiro-Wilk test, and then an independent sample t-test was employed. A p-value of 0.05 was regarded as significant.

## Results

**Figure 1:**
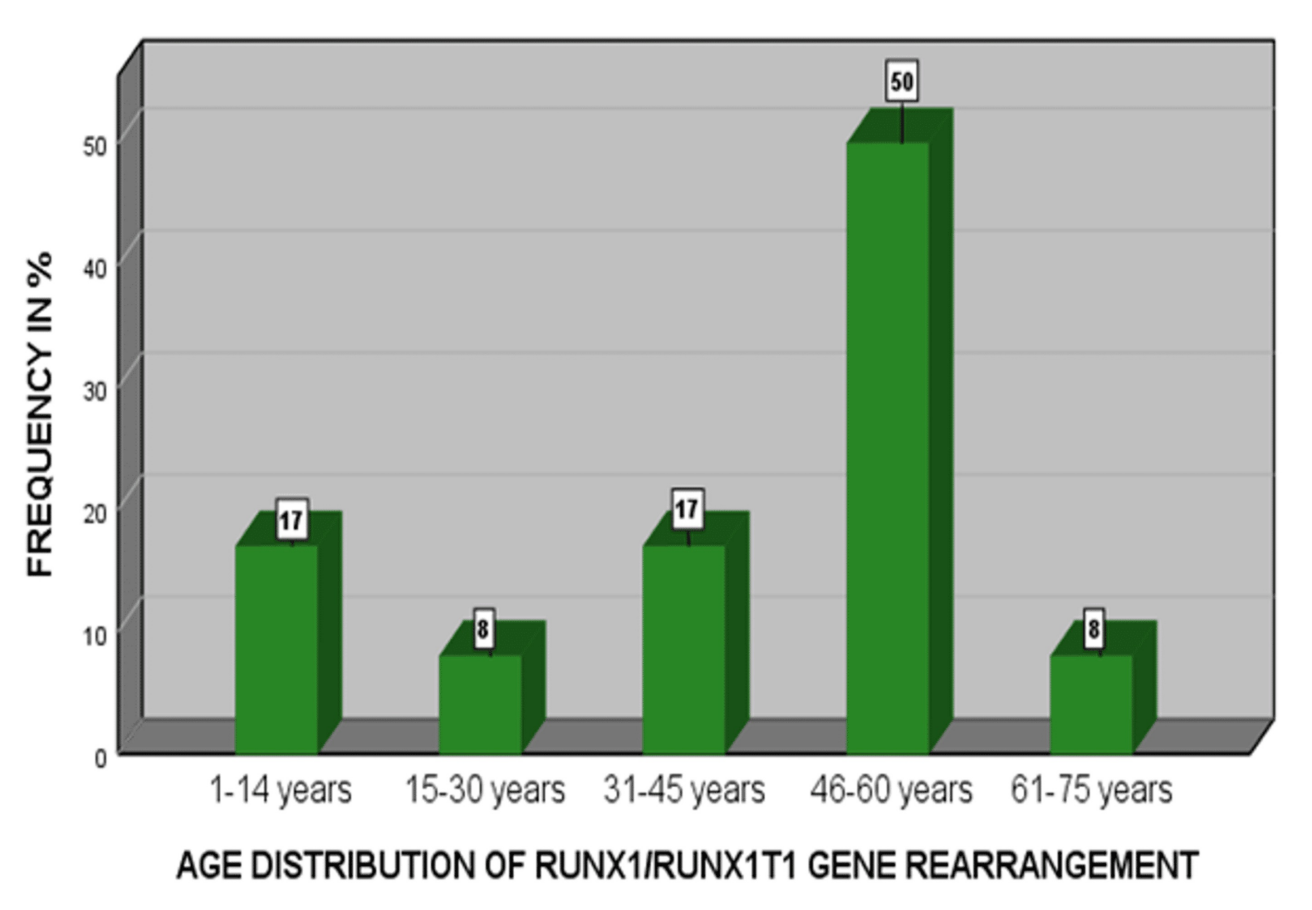
Age group-wise distribution of RUNX1/RUNX1T1 abnormality in acute myeloid leukemia (AML) patients

In the current study, 101 diagnosed AML cases were enrolled. Twelve (11.9%) patients presented as acute myeloblastic leukemia with maturation (AML-M2) FAB subtype of the total cases. The male-to-female ratio was 9:3, as 75% were male and 25% were female among the positive patients. The mean age of our study population was 42±1.288 years. The most common age group shown in this FAB subtype of AML-M2 with t(8;21) was between 46-60 years, with male predominance as shown in Figure 1.

The most common age group was 44-56 years, predominately male, with a p-value of 0.001, suggestive of significant occurrence in this age group when compared to other study groups. The demographic data are shown in Table 1.

**Table 1:** Demographic characteristics of participants with positive acute myeloblastic leukemia with maturation (AML-M2) with RUNX1/RUNX1T1

Characteristics	Frequency (%)	p-value
Gender		
Male	9 (75%)	0.003
Female	3 (25%)	
Age (years)		
5-17	1 (8%)	0.0001
18-30	3 (25%)	
31-43	2 (17%)	
44-56	5 (42%)	
57-69	1 (8%)	

The most common signs and symptoms in AML-M2 patients were pallor, fever, weight loss, gum, and nose bleeding, as shown in Table 2. The most common sign was pallor (83.3%, n = 10), followed by fever (75%, n = 9) and weight loss in 66.7% of patients (n = 8) with a p-value of 0.0001 on the application of the chi-square test. The least common manifestations were disseminated intravascular coagulation (DIC), bruises, and liver enlargement as outlined in Table 2.

**Table 2:** Clinical features of participants with positive acute myeloblastic leukemia with maturation (AML-M2) with RUNX1/RUNX1T1

Most common signs and symptoms	Frequency (n) and percentages (%)	p-value
Pallor	10 (83.3%)	0.0001
Weight loss	8 (66.7%)	
Splenomegaly	7 (58.3%)	
Palpitation	6 (50.0%)	
Bone pain	5 (41.6%)	
Infections	5 (41.6%)	
Lymph node enlargements	4 (33.3%)	
Fatigue	2 (16.7%)	
Bruises	1 (8.3%)	
Fever	9 (75.0%)	
Gum bleeding	7 (58.3%)	
Breathlessness	6 (50.0%)	
Body ache	6 (50.0%)	
Skin petechiae	5 (41.7%)	
Gums swelling	4 (33.3%)	
Nose bleeding	3 (25.0%)	
Liver enlargement	2 (16.6%)	
Disseminated intravascular coagulation (DIC)	1 (8.3%)	

The CBP of these positive patients showed leukocytosis, anemia, and thrombocytopenia. The mean, standard deviation, and ranges are represented in Table 3. There was marked leukocytosis in all the patients, with a mean value of 71.43 ± 37.93, a range of 35-150 × 10^9^/L, and a p-value of 0.0002 suggestive of marked leukocytosis in positive AML-M2 with RUNX1/RUNX1T1 patients. Similarly, there was marked thrombocytopenia in the study population with a p-value of 0.026 as explained in Table 3. There was marked pallor in the study population, as explained in Table 2, and it was confirmed by hemoglobin levels with a p-value of 0.001 suggestive of anemia as a cardinal feature in positive AML-M2 with RUNX1/RUNX1T1 patients. In differential leukocyte count (DLC), neutrophils ranged from 1% to 35%, and lymphocytes ranged from 40% to 96%.

**Table 3:** Mean standard deviation and ranges of hematological parameters

Hematological parameters	Mean + standard deviation	Ranges	p-value
Total leukocytes count (TLC)	71.43 ± 37.93	35-150×10^9^/L	0.0002
Platelets count (Plts)	41.5 ± 28.39	(7-93×10^9^/L)	0.026
Hemoglobin (Hb)	8.0 ± 2.25	(2-11g/dl)	0.001

**Figure 2:**
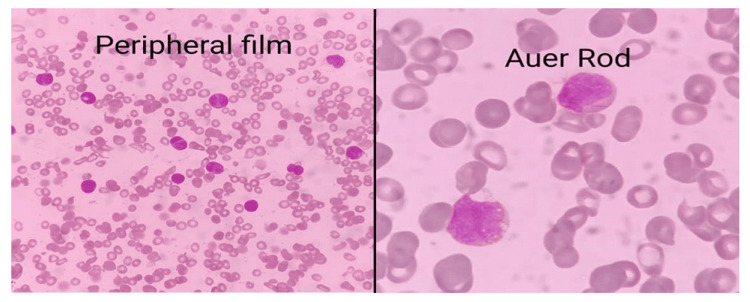
Peripheral and bone marrow examination of acute myeloid leukemia (AML) patients having RUNX1/RUNX1T1 or t(8;21) abnormalities

Bone marrow aspiration was also performed, which showed hypercellularity. Erythropoiesis, thrombopoiesis, and myelopoiesis were depressed in all positive cases of AML-M2. The blast cell size varied from medium to large with their percentages (50%-85%). Auer rods were also found in these patients on peripheral smears, as explained in Figure 2.

Bone marrow aspiration was performed, which showed hypercellularity. Erythropoiesis, thrombopoiesis, and myelopoiesis were depressed in all positive cases of AML-M2 as explained in Table 4.

**Table 4:** Morphological features of positive acute myeloblastic leukemia with maturation (AML-M2) patients

Morphological parameters	Frequency (n) and percentages (%)
Cellularity	
Hypercellular	12 (100%)
Hematopoiesis	
Depressed erythropoiesis	12 (100%)
Depressed myelopoiesis	12 (100%)
Reduced megakaryocytes	12 (100%)
Auer rods	12 (100%)
Size of blast cells (medium to large)	12(100%)

**Figure 3:**
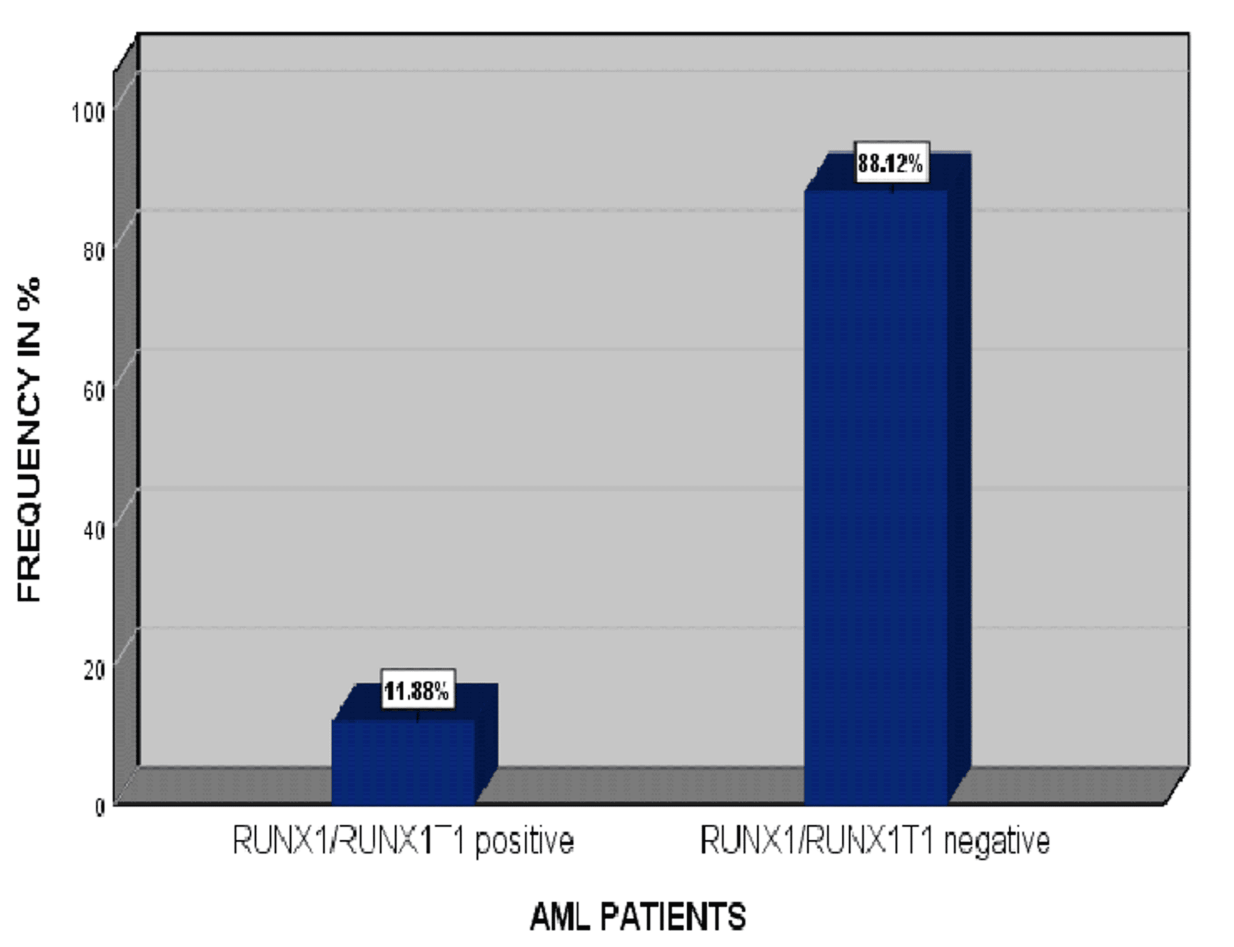
Relative frequency of positive RUNX1/RUNX1T1 gene rearrangement in acute myeloid leukemia (AML) patients

All positive cases of AML-M2 presented this RUNX1/RUNX1T1 gene rearrangement on RT-PCR, as shown in Figure 3.

## Discussion

In the present study, 101 patients were diagnosed with AML, and their consent was taken for research purposes only. A detailed clinical history and examination followed by a complete hematological workup, along with special stains and a bone marrow examination. The t (8;21) abnormality is found in approximately 5%-10% of all AML cases and 10%-22% of AML cases with maturation corresponding to the previous FAB class M2 [16]. However, in the current study, the total documented frequency of positive RUNX1/RUNX1T1 gene arrangement in AML patients was 11.9%.

The cure rate in AML patients depends on the patient's age and performance status [17]. The current study found that the most common age group that presented this specific chromosomal translocation t (8;21) was (44-56 years). This specific gene rearrangement occurrence is uncommon in patients aged over 60 years. Therefore, the incidence of AML with favorable cytogenetic abnormalities t(8;21) and recovery rate decreased with age.

The study by Panuzzo et al. [18] explains the clinical features of AML-M2 including anemia, fever, and bleeding. Some patients were prone to DIC, cerebral hemorrhage, and other serious complications, with high mortality rates. In the current study on AML-M2 positive patients, the main presenting symptoms were pallor in 10 (83.3%) and fever in nine (75.0%) cases, weight loss in eight (66.7%) cases, gums bleeding in seven (58.3%), breathlessness, palpitation, and body ache in six (50.0%), bone pains, infections, and skin petechiae in five (51.6%) cases, gums swelling in four (33.3%) cases, nose bleeding in three (25%) cases, fatigue in two (16.6%) cases, and bruises and DIC in one (8.3%) case.

It was observed in the present study that two (16.6%) patients had liver enlargement, splenomegaly was reported in seven (58.3%) patients, and four (33.3%) patients showed lymph node enlargement. It was also reported in a study by Qin et al. [7] that the specific chromosomal translocation t(8;21)(q22;22) was associated with hepatomegaly, splenomegaly, and lymphadenopathy. The study conducted by Qin et al. [7] also showed that the complete blood count (CBC) of patients with t(8;21)(q22;22) was significantly associated with thrombocytopenia (platelets ≤ 20 × 10^9^/L), anemia (hemoglobin ≤ 8 g/dl), and leukocytosis (total leukocyte count ≥ 20 × 10^9^/L) in adult patients, respectively. Similarly, in the current study, all the positive patients were presenting with anemia and thrombocytopenia. Hemoglobin and platelet counts ranged from 2-11 g/dl and 7-93 × 10^9^/L, with their mean hemoglobin and mean platelets being 8 g/dl and 42 × 10^9^/L, respectively. Total leukocyte count and erythrocyte sedimentation rate (ESR) were elevated and ranged between 35-150 × 10^9^/L and 12-90 mm/hour, respectively.

Another study was conducted by Lei et al. [14], which reported that in AML patients there were 85% FAB AML-M2, 8% AML-M1, and 7% AML-M4 subtypes. Prominent morphological characteristics included dysplasia in maturing myeloid cells (83%), long thin tapered Auer rods and cytoplasmic vacuoles (58%), eosinophilia (50%), and mast cells (22%). In the current research, these positive patients presented morphological features related to AML-M2. The process of hematopoiesis was depressed in all positive AML-M2 patients. The size of blast cells varied from medium to large size, ranging from 30% to 90% with the presence of Auer rods. The mean AML-M2 blast percentage was 58.91%.

Acute myeloid leukemia patients with t(8;21) have a good prognosis. Most of the patients achieve complete remission after induction therapy. The overall survival rate is about 60% [19]. In the current study, the diagnosis of this gene rearrangement helped in patient management. 

## Conclusions

The present study highlights that the microscopic features of peripheral smear and bone marrow examination remain vital for the diagnoses of the AML-M2 subtype in AML patients. Molecular analysis helps in disease categorization. Our study suggests that detecting t(8;21) with RUNX1/RUNX1T1 gene rearrangement is essential for the clinical decision-making of these patients, as it has both prognostic and therapeutic implications.
